# Lead Borate Glasses and Glass-Ceramics Singly Doped with Dy^3+^ for White LEDs

**DOI:** 10.3390/ma13215022

**Published:** 2020-11-07

**Authors:** Agata Górny, Marta Kuwik, Wojciech A. Pisarski, Joanna Pisarska

**Affiliations:** Institute of Chemistry, University of Silesia, Szkolna 9 Street, 40-007 Katowice, Poland; marta.kuwik@us.edu.pl (M.K.); wojciech.pisarski@us.edu.pl (W.A.P.)

**Keywords:** lead borate glasses, glass-ceramics, dysprosium ions, white luminescence

## Abstract

In this paper, some series of lead borate glasses and glass ceramics singly doped with Dy^3+^ ions were prepared and then studied using spectroscopic techniques. Our research includes mainly studies of the luminescence properties of received materials for white light. The luminescence bands associated with the characteristic ^4^F_9/2_→^6^H_15/2_ (blue), ^4^F_9/2_→^6^H_13/2_ (yellow) and ^4^F_9/2_→^6^H_11/2_ (red) transitions of trivalent dysprosium in lead borate systems are well observed. In particular, the Commission Internationale de I’Eclairage (CIE) chromaticity coordinates (x, y) were calculated in relation to potential applications for white light-emitting diodes (W-LEDs). Their values depend on the relative B_2_O_3_/PbO ratios and PbX_2_ contents (where X = Cl, F, Br) in glass composition. For glass-ceramics, the chromaticity coordinates are changed significantly under different excitation wavelengths.

## 1. Introduction

Rare earth doped inorganic glasses are promising amorphous materials for tricolor W-LEDs (white light emitting diodes), solid-state lasers emitting visible light or near-infrared radiation, and other optoelectronic device applications [1,2,3,4,5]. In particular, white light-emitting devices have attracted considerable interest for their use in diodes and liquid crystal monitor screens. In this field, inorganic glasses are particularly studied for generating white luminescence [6,7,8,9]. Different glass matrices were tested in order to find the best materials emitting white light. In particular, borate glasses containing various glass-network modifiers and rare earth ions acting as optically-active ions have aroused considerable attention for white-emitting laser sources.

Borate based glasses are excellent amorphous materials for numerous scientific and industrial applications [10]. Among the different oxide components, such as SiO_2_, GeO_2_ or P_2_O_5_, successfully used for glass synthesis, B_2_O_3_ is the best glass-former, which makes glass with high chemical durability and thermal stability, good transparency and rare earths solubility. Moreover, borate-based glasses mixed with different modifier oxides [11] and halides [12] have attractive optical and structural properties. These materials containing B_2_O_3_ are promising systems for optoelectronic devices, lasers and white light applications [13,14,15,16,17,18,19]. Simultaneously, boron oxide can be incorporated with either Bi_2_O_3_ [19] or PbO [20], with the dominant percent forming chemically and thermally stable glasses. Great attention has been paid to lead borate glasses due to their distinct advantages, including good radiation shielding properties, strong absorption in the UV region, large transmission windows and the relatively large refractive indices compared to pure borate glasses or alkali borate glasses [21,22]. Recent studies indicate that the properties of systems based on PbO–B_2_O_3_ containing rare earth ions are significant in the field of solid-state lasers, tri-color LEDs, reflecting windows and optoelectronic devices [23,24,25,26,27,28].

Among numerous rare earth-doped host materials, the inorganic Dy^3+^-doped glasses are really important systems for developing solid state lasers operating in the visible region [29]. Various lead-free [30,31,32,33,34] and lead-based [35,36,37,38,39,40] glass systems singly doped with dysprosium ions are also widely used to generate white light due to their simultaneous blue, yellow and red emissions. This behavior is associated with transitions originating from the state ^4^F_9/2_ to the lower lying states ^6^H_J/2_ (J = 11, 13, 15). Based on the integrated intensities of the emission bands related to the ^4^F_9/2_→^6^H_15/2_ (blue) and ^4^F_9/2_→^6^H_13/2_ (yellow) transitions of trivalent dysprosium ions, the yellow to blue fluorescence intensity ratio can be calculated. The ratio Y/B (yellow to blue) for dysprosium ions is an important spectroscopic factor in evaluating the studied system for white light generation. What is more, dysprosium-doped borate-based glasses belong to promising matrices that can be used to generate white light [41,42,43,44,45,46,47], for which the ratio Y/B (Dy^3+^) oscillates within the limits of one. In general, oxide and oxyfluoride borate-based glasses with Dy^3+^ have become one of the most important classes of materials because of their potential application in numerous fields, but nowadays most of the studies are concentrated on generating white light.

Furthermore, lead borate-based glasses containing rare earth ions belong to interesting luminescent amorphous materials. The optical properties of glasses based on PbO-B_2_O_3_-Al_2_O_3_-WO_3_-Ln_2_O_3_ (Ln—rare earth ions) without or with PbX_2_ (X = Cl, Br or F) were presented and discussed in the reviewed work published by us ten years ago [48]. Structural and optical aspects for Ln^3+^ ions have been demonstrated in glass systems containing PbF_2_ [49]. The interesting results were also obtained for Dy^3+^-doped lead borate glass, where the stimulated emission cross-section (σ_em_ = 5.57 × 10^−21^ cm^2^ at λ_p_ = 573 nm) as well as the quantum efficiency (η = 70%) for the main ^4^F_9/2_→^6^H_13/2_ yellow transition are relatively large, suggesting its potential solid-state laser application [50]. Multicomponent glasses yPbX_2_-(1-y)PbO-B_2_O_3_-Al_2_O_3_-WO_3_-Dy_2_O_3_ were also thermally treated to fabricate glass-ceramic materials containing the nano- or microcrystal PbX_2_ (where X = Cl, Br or F), owing to its larger tendency to crystallize lead halide, especially lead fluoride, than oxides. Unexpectedly, the results obtained by us were completely different. Independently on PbX_2_, PbWO_4_ microcrystals with tetragonal unit cells (PDF-2 card no. P190708) were successfully formed in transparent lead borate glass-ceramics doped with Dy^3+^ ions [51].

This paper shows the results for lead borate glasses and glass-ceramics containing Dy^3+^ ions, and the applicability of these systems as white light emitters. Glass samples with Dy^3+^ were prepared, and in the next step, they were heat-treated in order to obtain transparent glass-ceramics. Based on the emission spectra measured for dysprosium ions in glasses and glass-ceramics, the chromaticity coordinates (x, y) were calculated in relation to their potential application in W-LEDs.

## 2. Materials and Methods

Glass systems singly doped with dysprosium ions containing various B_2_O_3_/PbO ratios were prepared by the traditional melt quenching technique from high-purity raw materials (99.99% Aldrich Chemical Co., St. Louis, MO, USA). The B_2_O_3_/PbO ratios are 2:1, 1:1, 1:2, 1:4, 1:5 and 1:8. Each glass composition in wt.% is presented in Table 1.

During synthesis, the appropriate amounts of metal oxides were mixed in a glow-box in an atmosphere of dried argon and placed in a corundum crucible. The samples with B_2_O_3_/PbO ratios 1:4, 1:5 and 1:8 were melted at 850 °C, whereas the systems with B_2_O_3_/PbO ratios 2:1, 1:1 and 1:2 were melted for 1 h at 1250 °C.

Furthermore, glasses with 9 wt.% PbX_2_ (where X = Cl, F or Br) were synthesized for a 2:1 system of B_2_O_3_/PbO. Lead oxide was partially replaced by PbX_2_. To prepare glass samples, metal halides and metal oxides were mixed together in an agate ball mill. Similar to the oxide glass system, the samples were fabricated in a glow-box in an atmosphere of dried argon. The samples were melted in a corundum crucible at 850 °C for 1 h. From the prepared glass systems, the glass-ceramics were obtained. In order to fabricate transparent glass-ceramic materials, the lead borate glass systems with PbX_2_ (X = Cl, F, Br) were annealed at 450 °C for 5–15 h. Independently on PbX_2_, lead tungstate PbWO_4_ microcrystals with tetragonal unit cells were successfully formed in transparent lead borate glass-ceramic materials containing dysprosium ions.

Measurements were carried out on a PTI QuantaMaster QM40 spectrofluorimeter (Photon Technology International, Birmingham, NJ, USA) coupled with the optical parametric oscillator (OPO), pumped by the third harmonic of a Nd:YAG laser (OpotekOpolette 355 LD, OPOTEK, Carlsband, CA, USA). Double 200 mm monochromators were used to detect luminescence spectra. Then, the spectra with ±0.1 nm resolution were recorded using a multimode detector UVVIS PMT (R928). Decay curves were registered by a PTI ASOC-10 (USB-2500) oscilloscope with an accuracy of ±1 µs. The Raman spectra were measured using a Thermo Fisher Scientific^TM^ DXR^TM^2xi Raman Imaging Microscope (Thermo Scientific, Waltham, MA, USA). The appropriate laser source with an excitation wavelength of 780 nm was used to obtain the Raman spectra. Measurements were performed at room temperature.

## 3. Results and Discussion

### 3.1. Glasses

**Figure 1:**
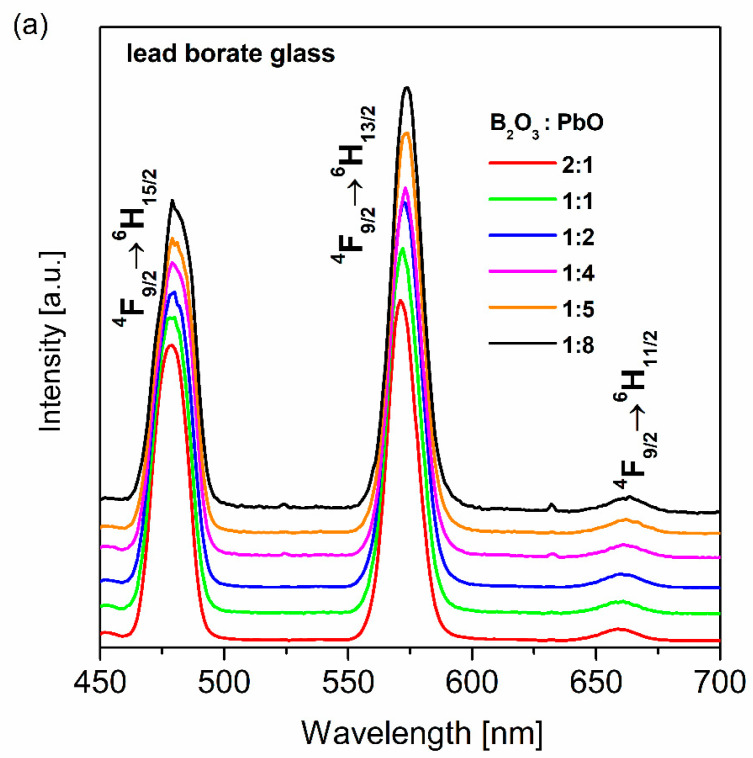

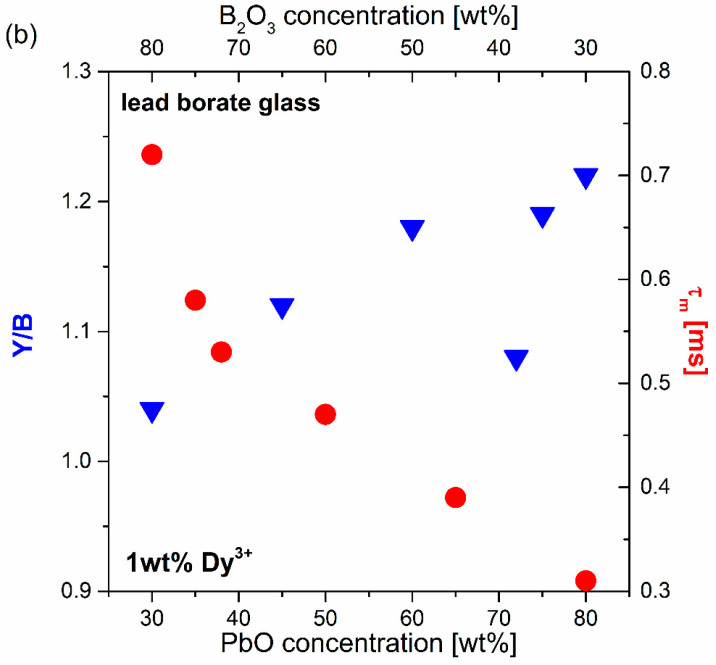
Emission spectra for dysprosium-doped lead borate glass systems with different B_2_O_3_:PbO ratios (**a**), and the influence of B_2_O_3_:PbO ratio on spectroscopy parameters for dysprosium ions (**b**).

Figure 1a shows the emission spectra for glass systems singly doped with dysprosium ions with different B_2_O_3_:PbO weight ratios.

Independently on the excitation wavelengths 390 nm (^4^K_17/2_ level) and 450 nm (^4^I_15/2_ level), the spectrum consists of luminescence bands characteristic for Dy^3+^ ions. The spectra exhibit strong blue and yellow emission bands, which are assigned to the ^4^F_9/2_→^6^H_15/2_ and ^4^F_9/2_→^6^H_13/2_ electronic transitions of dysprosium, respectively. Moreover, a significantly weaker red emission band corresponding to the ^4^F_9/2_→^6^H_11/2_ transition is noticeable.

Figure 1b shows the dependence of the luminescence intensity ratios Y/B, related to the integrated emission intensities of ^4^F_9/2_→^6^H_13/2_ (yellow) and ^4^F_9/2_→^6^H_15/2_ (blue) transitions and the measured lifetimes for the ^4^F_9/2_ excited state of Dy^3+^, on B_2_O_3_:PbO ratios. Both spectroscopic parameters depend strongly on the amount of PbO in the glass composition. These aspects were examined in detail in the previously published work [52], and they are not discussed here. Among rare earths, materials doped with dysprosium ions, such as inorganic glass systems, are found to be an appropriate candidate for white light applications. Dysprosium ions reveal two intense luminescence bands in the blue and yellow spectral regions. These bands correspond to the ^4^F_9/2_→^6^H_15/2_ and ^4^F_9/2_→^6^H_13/2_ transitions, as mentioned above. The proper combination of yellow and blue luminescence bands can produce white light. The yellow to blue luminescence intensity ratio can be adjusted by varying the glass-host [53], the pumping wavelengths [54] and the dysprosium ion concentration [55].

The ratio Y/B is influenced by the effect between the dysprosium ions and the nearest surroundings. In general, the yellow emission of Dy^3+^ ions is stronger if the Y/B factor is higher. Therefore, near-white light luminescent materials are possible obtainable by adjusting the yellow to blue intensity ratio of dysprosium. When the Y/B ratio is greater than unity, the degree of covalent character between the dysprosium ions and the surrounding ligands is higher, suggesting that the nature of the host environment is more disordered. Our previous experimental results clearly shown that the yellow to blue intensity ratio values decreased from 1.59 for lead germanate glass to 1.12 for lead borate glass [56]. Moreover, the Y/B ratio of the dysprosium ions changes with the reduction in the covalent character of heavy metal oxides in the following direction: GeO_2_→SiO_2_→P_2_O_5_→B_2_O_3_. Therefore, the lead borate glass-host was chosen for further research into W-LED applications. The studies on lead borate glass evidently show that the Y/B ratio of trivalent dysprosium ions seems to be 1.04 when the concentration of B_2_O_3_ is the highest (B_2_O_3_:PbO = 2:1), which is favorable for white light applications. The interesting results for lead borate glasses were also obtained in previous studies conducted by Jayasankar et al. [57], which confirm that the yellow to blue factor is more sensitive for glass hosts with lower Dy^3+^ ion concentrations.

In order to evaluate white light chromaticity coordinates, a CIE diagram is usually employed. This is regulated by the Commission Internationale de l’Éclairage, defined in 1931, and it expresses all colors by using the three primary colors of X, Y, and Z. The chromaticity color coordinates (x, y) can be calculated from the emission spectra [58].

**Figure 2:**
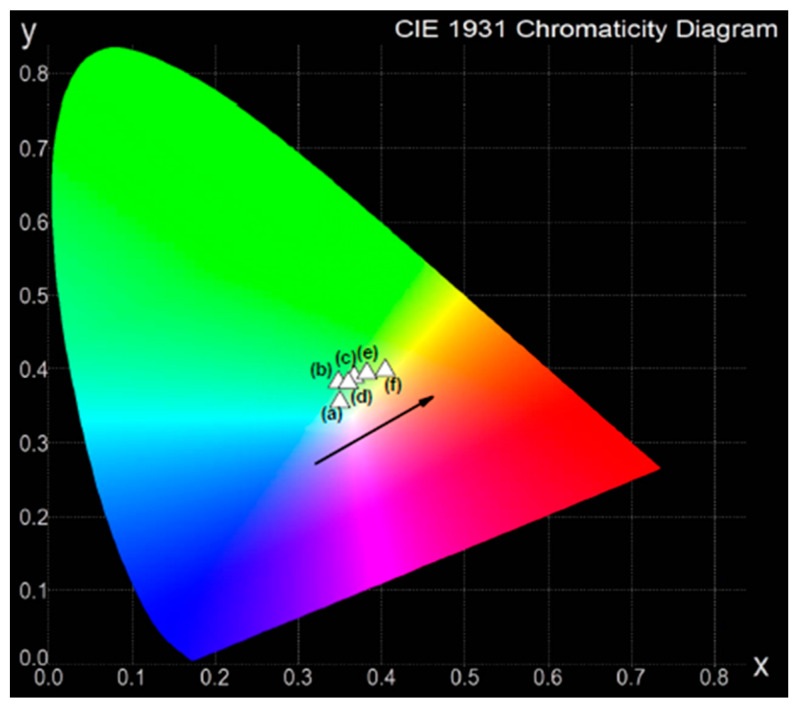
Chromaticity coordinates in the CIE diagram for Dy^3+^-doped lead borate glasses with different B_2_O_3_:PbO relative ratios.

The chromaticity color coordinates (x, y) of lead borate glass systems containing various B_2_O_3_/PbO weight ratios were calculated. The obtained values are given in Table 2. The coordinates for the glass samples singly doped with dysprosium ions are located in the green and green–yellow region of the CIE diagram shown in Figure 2. The presence of PbO in the glass systems causes the color coordinates to shift from the green region to the greenish–yellow for samples with the highest (80 wt.%) amount of lead oxide. However, the exception is the sample containing the highest amount of B_2_O_3_. In this case, the chromaticity coordinates are located nearest to the white point in the CIE diagram. Therefore, introducing PbO into the glass host is rather unfavorable. The less lead oxide there is in the glass systems, the closer the chromaticity coordinates approach to white. Moreover, glass materials containing Pb^2+^ ions are often eliminated because of their well-known hazardous effects. On the other hand, the systematic luminescent and energy transfer studies indicate that dysprosium-doped lithium lead alumino borate glasses are promising candidates for W-LED and laser applications [59].

**Figure 3:**
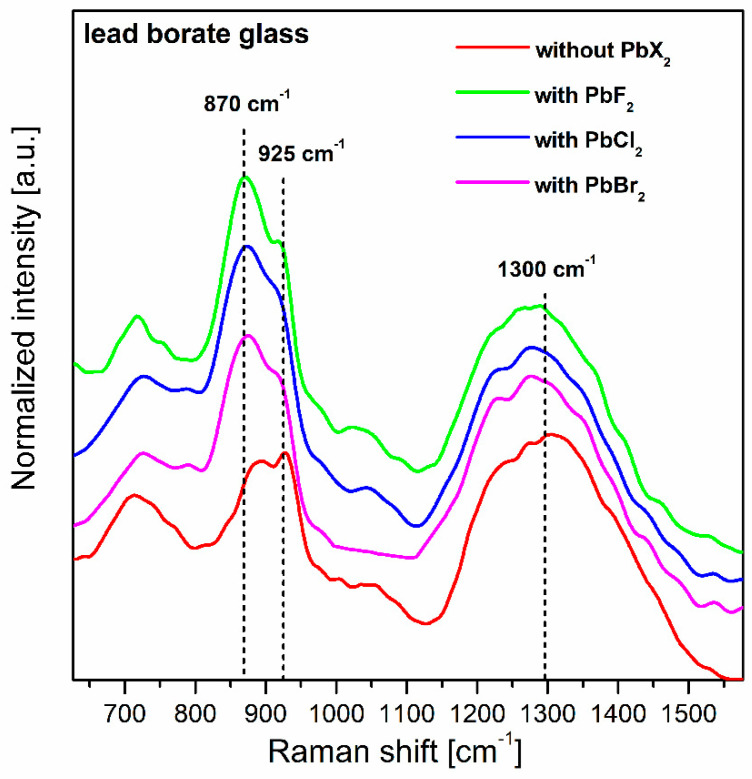
The Raman spectra for lead borate glasses with PbX_2_ (X = Cl, Br, F).

In order to gradually eliminate the lead oxide from the glass-host, lead halide borate glasses were synthesized. In this case, lead oxide was partially substituted with PbX_2_ (X = Cl, Br, F). At this moment, it should be mentioned that for mixed oxyhalide systems, the nominal starting composition and final glass composition are rather different due to the gases lost during the melting process, according to the chemical reaction PbX_2_ + H_2_O → PbO + 2HX↑. Previously published works suggest that the losses of fluorine or chlorine components increase when the PbX_2_ content in the glass composition is the greatest [60,61,62]. The presence of PbX_2_ in the mixed lead halide borate glasses was also evidenced by the Raman spectra measurements. Figure 3 presents the Raman spectra for glass samples in the absence and presence of PbX_2_ (X = Cl, Br, F).

In general, the local structure of lead borate glass was changed, from trigonal BO_3_ to pentaborate groups containing three- and four-coordinated boron atoms, when PbX_2_ was added to the base glass. This results in Raman bands at about 870 cm^−1^ and 925 cm^−1^, because of the greater number of borate units containing non-bridging oxygen (NBO). The Raman investigations clearly indicate that the intensities of these vibration bands associated with the BO_4_ units and NBO atoms increase with the presence of PbX_2_ (X = Cl, Br, F). They are assigned to the pentaborate groups [63]. Similar results were obtained earlier for the B_2_O_3_–BaF_2_–LiX (X denotes F, Cl or Br) glass systems [64]. The Raman bands near 1300 cm^−1^ are related to the maximal phonon energy of the glass host. For our lead borate glasses, the Raman band is decreased from 1300 cm^−1^ (glass without PbX_2_) to nearly 1277 cm^−1^ (glass with PbX_2_), suggesting the important role of lead halide in the formation of the glass host. This was also confirmed by thermal measurements and the calculation of bonding parameters from the absorption spectra of lead borate glasses doped with dysprosium ions [65]. The glass transition temperature T_g_ for glass without PbX_2_ is close to 440 °C, and this value is reduced for system with PbX_2_ (X = Cl, Br, F) in direction Br (390 °C) → Cl (375 °C) → F (340 °C), respectively. Similar effects were obtained for the bonding parameter δ. Its value can be positive or negative, suggesting the existence of more covalent or more ionic bonding between Dy^3+^ ions and their nearest surrounding. For lead borate glass system without PbX_2_, the bonding parameter is equal to δ = −0.77, indicating the ionic relation between rare earths and ligands.

The values of δ decrease when samples containing PbX_2_ (X = Cl, Br or F) are changed in the direction Br (−0.90) → Cl (−0.94) → F (−1.04). This indicates that the environment around the active ions (Dy^3+^) is more ionic [65]. Further studies revealed that the coordination sphere around the optically active ions (Er^3+^) is changed significantly when lead halide is introduced to the lead borate glass. The spectral bandwidth (FWHM) for the main ^4^I_13/2_→^4^I_15/2_ near-infrared laser transition of Er^3+^ is reduced in the following direction: Br → Cl → F. This suggests evidently that the glass structure around the optically active ions was changed during the substitution of PbO by PbX_2_, and the fraction of halide ions was successfully bridged with Er^3+^ [66].

**Figure 4:**
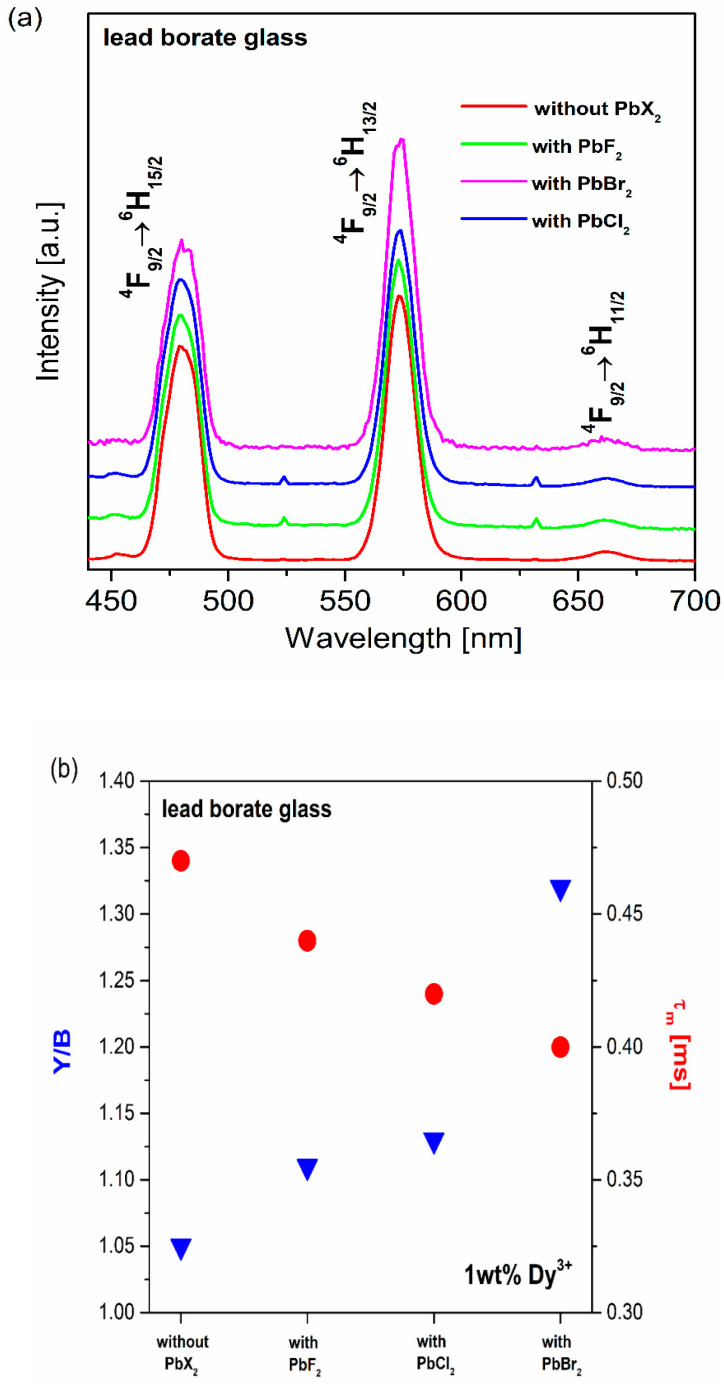
Emission spectra for dysprosium-doped lead borate glass systems containing PbX_2_ (**a**). influence of PbX_2_ (X = Cl, Br, F) on spectroscopy parameters for dysprosium ions (**b**).

**Figure 5:**
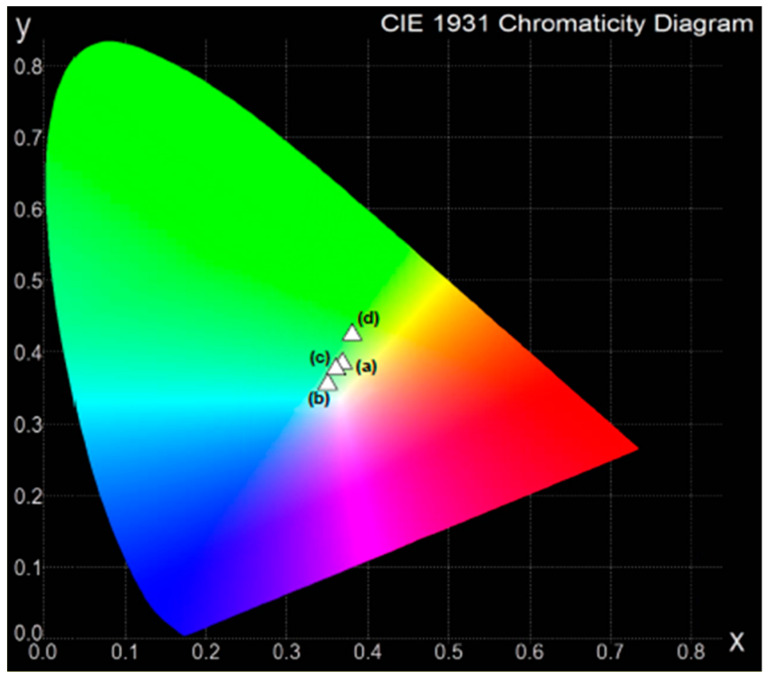
Chromaticity coordinates in CIE diagram for Dy^3+^-doped lead borate glass with PbX_2_ (X = Cl, Br, F).

Figure 4a presents the emission spectra for lead borate glasses containing lead halide and Dy^3+^ ions. Figure 4b shows schematically the Y/B factors and the measured lifetimes of ^4^F_9/2_ (Dy^3+^) for samples without or with PbX_2_ (X = Cl, Br, F). The spectra were also excited at 450 nm, and consist of emission bands characteristic of transitions of Dy^3+^ ions. Interestingly, all the bands exhibit an increase in emission intensity when the halide glass-modifiers are increased. However, from the perspective of white light emission, the most interesting is the ratio of yellow to blue bands. Our investigations indicate that the emission intensities of blue and yellow bands depend on the halide glass-modifier. From the calculated values, it was found that the yellow to blue factor of Dy^3+^ for glass samples containing lead fluoride is the best, and is near one (Y/B = 1.11), which confirms the possibility of white light emission. Similar results have also been described by Babu et al [67], whereby a Y/B factor equal to 1.05 was received for the oxyfluoride glass sample containing 1% dysprosium ions. For lead borate glass system with PbX_2_ (X = Cl, Br, F), the chromaticity coordinates (x, y) were also calculated. Table 3 presents all the results. Furthermore, the chromaticity coordinates show the location of the emission color in the chromaticity diagram given in Figure 5. Among the prepared samples, the lead borate glass with PbF_2_ presents CIE values nearest to the chromaticity coordinates of perfect white light (0.333, 0.333). In conclusion, it can be stated that lead borate glass systems with PbF_2_ are potentially useful for white light emission.

### 3.2. Glass-Ceramics

Controlled heat treatment elicits the transformation process from precursor glasses to transparent samples of glass-ceramics (TGC). During the thermal treatment of mixed oxyfluoride systems, fluoride micro- or nanocrystals are usually formed. They are dispersed into oxide amorphous matrices. Rare earth ions are usually distributed in both crystalline and amorphous phases. This was presented for several transparent glass-ceramic materials [67,68,69,70]. The heat treatment process was also applied for lead borate glasses. With a controlled annealing time and temperature, lead borate glass-ceramic materials containing PbWO_4_ microcrystals and dysprosium ions were successfully fabricated and then characterized using various experimental techniques [67].

**Figure 6:**
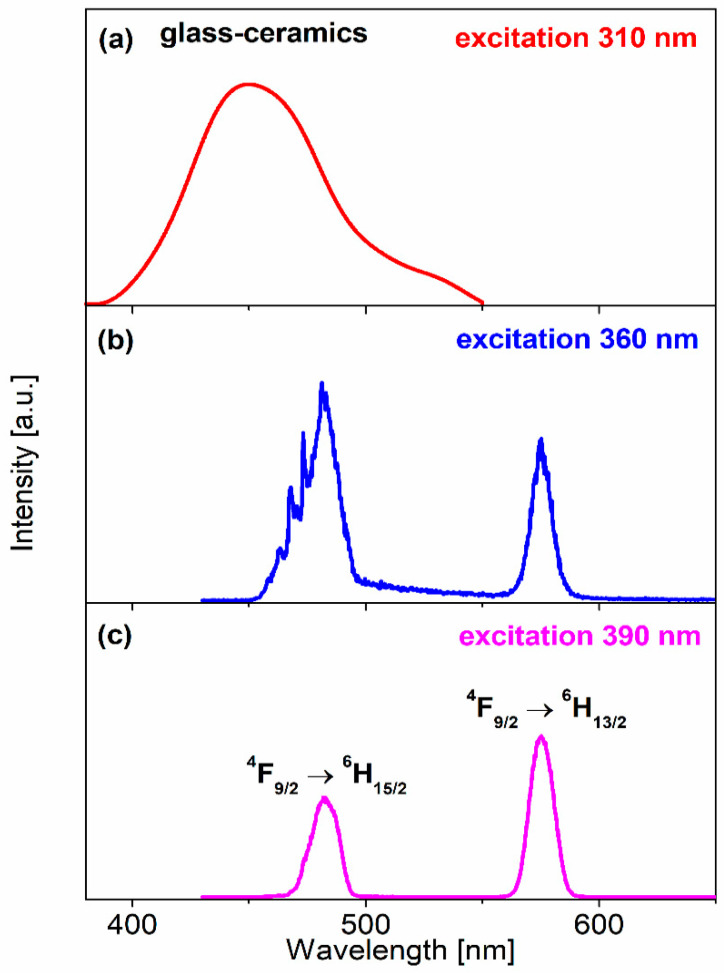
Emission spectra for Dy^3+^ doped lead borate glass-ceramic materials under the various excitation wavelengths (**a**) 310 nm, (**b**) 360 nm and (**c**) 390 nm.

Figure 6 shows the emission spectra for lead borate glass-ceramics singly doped with dysprosium ions, which were measured under the various excitation wavelengths 310 nm, 360 nm and 390 nm.

**Figure 7:**
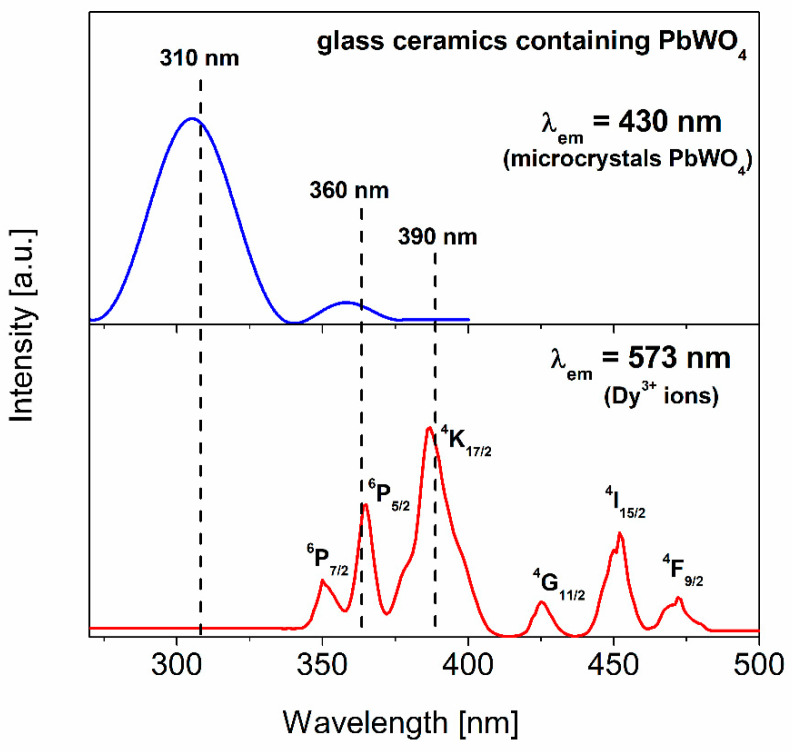
The excitation spectra measured for the studied glass-ceramic materials.

The spectrum obtained under excitation λ_exc_ = 310 nm consists of a broad emission band centered at 450 nm, which is characteristic for the crystalline phase PbWO_4_ [71,72]. With the excitation of the glass-ceramic sample at 360 nm or 390 nm, the observed bands located at about 480 nm and 573 nm are characteristic for Dy^3+^ ions, and correspond to the ^4^F_9/2_→^6^H_15/2_ (blue) and ^4^F_9/2_→^6^H_13/2_ (yellow) transitions, respectively. The relative luminescence intensities of the blue and yellow bands strongly depend on the excitation wavelengths 360 nm and 390 nm. In particular, the blue emission band, related to the ^4^F_9/2_→^6^H_15/2_ transition of dysprosium, is enhanced significantly when the glass-ceramic sample is directly excited at 360 nm. It can be inferred that the enhanced intensity of the blue band is the result of the superimposition of the PbWO_4_ microcrystals present in the glass-ceramic system and Dy^3+^ luminescence (details are given in [73]). This was also confirmed by the excitation spectra measurements. Figure 7 presents the excitation spectra recorded for glass-ceramics containing PbWO_4_.

When the spectrum was monitored at λ_em_ = 430 nm, an intense peak centered at about 310 nm is well observed. It is assigned to an exciton excitation, and strictly related to the presence of crystalline phase PbWO_4_ [74]. The spectrum monitored at λ_em_ = 573 nm consists of six bands, which originate from the transition from the ^6^H_15/2_ ground state of dysprosium ions to the following levels: ^4^F_9/2_, ^4^I_15/2_, ^4^G_11/2_, ^4^K_17/2_ and ^6^P_J/2_ (J = 5 and 7). It is clearly seen that both the excitation bands due to the PbWO_4_ microcrystals and Dy^3+^ ions exist in the spectral range near 360 nm. Thus, the blue luminescent band corresponding to the ^4^F_9/2_→^6^H_15/2_ transition is enhanced and broadened, because PbWO_4_ crystallites and Dy^3+^ ions in the glass-ceramic sample are simultaneously excited at 360 nm.

In order to further discuss the potential application of the prepared glass-ceramic material in white light-emitting diodes, the chromaticity color coordinates are determined, and the results are given in Table 4. All the chromaticity coordinates for the TGC system excited at 310 nm, 360 nm and 390 nm were calculated from the luminescence spectra. The results for lead borate glass-ceramic materials revealed that the calculated chromaticity coordinates are completely different, depending critically on the excitation wavelengths.

**Figure 8:**
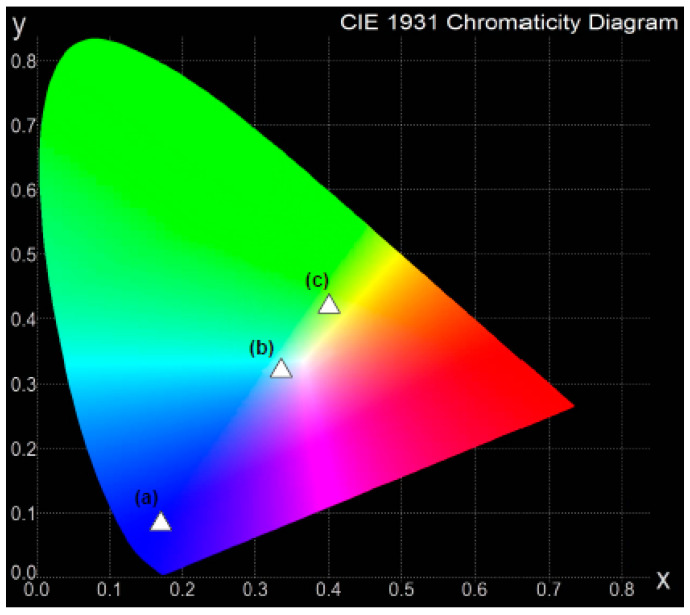
Chromaticity coordinates in CIE diagram for Dy^3+^ doped lead borate glass-ceramics under different excitation wavelengths.

Figure 8 presents the calculated chromaticity coordinates for glass-ceramics. They shifted from blue (λ_exc_ = 310 nm) and yellow–green (λ_exc_ = 390 nm) to bluish–white light when the glass-ceramic sample was directly excited at 360 nm. Similar effects of excitation wavelengths on emission intensities and CIE coordinates have been observed for SiO_2_-KYF_4_ nano-glass-ceramics [75]. Kemere et al. [76] concluded that the chromaticity coordinates of the aluminosilicate oxyfluoride glass-ceramic materials co-doped with Dy^3+^/Eu^3+^ are not changed drastically, but the heat treatment process of precursor glasses can be effectively used to tune the color of the emitted light. Our studies suggest that lead borate glass-ceramics containing dysprosium ions are attractive for multicolor luminescence applications. These TGC materials can be used in optoelectronic devices, such as light-emitting diodes. In particular, they may be sources of white emission when pumped directly at 360 nm.

## 4. Conclusions

To summarize, lead borate glasses and glass-ceramics singly doped with dysprosium ions were successfully synthesized via the melt-quenching technique. The received materials were studied using luminescence spectroscopy to confirm the possibility of white emission. It was proven that the Y/B factor of trivalent dysprosium ions for a system with the highest concentration of B_2_O_3_ (B_2_O_3_:PbO = 2:1) is close to 1.00. Moreover, the Y/B ratio value in the studied samples with PbX_2_ (X = Cl, Br, F) decreased from 1.32 to 1.11 with increases in the ionic character of lead halides from PbBr_2_ to PbF_2_. For all glass and glass-ceramic systems, the CIE values were calculated from the emission spectra in relation to the application of W-LEDs. The analysis of the CIE diagram indicates that the presence of lead oxide in borate glasses causes the color coordinates to shift from the green region to greenish–yellow for systems with the highest amounts of PbO. Among the prepared samples with PbX_2_, lead borate glass containing PbF_2_ presents CIE values nearest to the color coordinates of perfect white light. Furthermore, the tendency to shift the chromaticity coordinates from blue (λ_exc_ = 310 nm) and yellow–green (λ_exc_ = 390 nm) to bluish–white light was observed when the glass-ceramic sample was directly excited at 360 nm. Our studies demonstrate that the spectroscopic properties and chromaticity coordinates of lead borate systems can be effectively controlled by modification of the chemical composition of glass-hosts and changing the excitation wavelengths. There is an efficient way to obtain white light-emitting glasses and glass-ceramics. Based on the experimental and calculated results, we suggest the possibility of using these materials containing Dy^3+^ ions for future applications in white light generation.

## Figures and Tables

**Table 1:** Chemical compositions of Dy^3+^ singly doped lead borate glasses.

Glass No.	Ratio	Chemical Composition (wt.%)				
	B_2_O_3_:PbO	B_2_O_3_	PbO	Al_2_O_3_	WO_3_	Dy_2_O_3_
(a)	2:1	60	30	6	3	1
(b)	1:1	45	45	6	3	1
(c)	1:2	30	60	6	3	1
(d)	1:4	18	72	6	3	1
(e)	1:5	15	75	6	3	1
(f)	1:8	10	80	6	3	1

**Table 2:** The CIE (chromaticity coordinates) value and the Y/B factor for Dy^3+^ ions in the studied glass samples with different B_2_O_3_:PbO ratios.

Glass No.	Ratio	Y/B	CIE (x, y)
	B_2_O_3_:PbO		
(a)	2:1	1.04	(0.321, 0.352)
(b)	1:1	1.12	(0.319, 0.379)
(c)	1:2	1.18	(0.338, 0.386)
(d)	1:4	1.08	(0.331, 0.378)
(e)	1:5	1.19	(0.353, 0.391)
(f)	1:8	1.22	(0.375, 0.395)

**Table 3:** The chromaticity coordinates (x, y) for the Dy^3+^ ions in the studied glass samples containing PbX_2_ (where X = Cl, Br or F).

Glass No.	PbX_2_	Y/B	CIE (x, y)
(a)	without	1.04	(0.336, 0.381)
(b)	PbF_2_	1.11	(0.318, 0.352)
(c)	PbCl_2_	1.13	(0.328, 0.373)
(d)	PbBr_2_	1.32	(0.348, 0.421)

**Table 4:** The chromaticity coordinates (x, y) for Dy^3+^ ions in the studied glass-ceramic system under various excitation wavelengths.

Glass-Ceramics No.	λ_exc_ (nm)	CIE (x, y)
(a)	310	(0.142, 0.081)
(b)	360	(0.305, 0.317)
(c)	390	(0.370, 0.416)
